# Behavioral and neural evidence of enhanced long-term memory for untrustworthy faces

**DOI:** 10.1038/s41598-019-55705-7

**Published:** 2019-12-16

**Authors:** Mathias Weymar, Carlos Ventura-Bort, Julia Wendt, Alexander Lischke

**Affiliations:** 1 Department of Biological Psychology and Affective Science, University of Potsdam, Potsdam, Germany; 2 Department of Biological and Clinical Psychology, University of Greifswald, Greifswald, Germany

**Keywords:** Long-term memory, Social neuroscience

## Abstract

In daily life, we automatically form impressions of other individuals on basis of subtle facial features that convey trustworthiness. Because these face-based judgements influence current and future social interactions, we investigated how perceived trustworthiness of faces affects long-term memory using event-related potentials (ERPs). In the current study, participants incidentally viewed 60 neutral faces differing in trustworthiness, and one week later, performed a surprise recognition memory task, in which the same old faces were presented intermixed with novel ones. We found that after one week untrustworthy faces were better recognized than trustworthy faces and that untrustworthy faces prompted early (350–550 ms) enhanced frontal ERP old/new differences (larger positivity for correctly remembered old faces, compared to novel ones) during recognition. Our findings point toward an enhanced long-lasting, likely familiarity-based, memory for untrustworthy faces. Even when trust judgments about a person do not necessarily need to be accurate, a fast access to memories predicting potential harm may be important to guide social behaviour in daily life.

## Introduction

During social interactions, individuals automatically form impressions of others based on facial cues. These impressions, which mainly rely on valence evaluation, reflected by trustworthiness judgments^1^, occur rapidly^2^ and unintentionally^3,4^ and have significant consequences for social behavior. For instance, individuals with untrustworthy faces have lower chances to have their loans funded^5^ and are more likely to be sentenced for a crime^6^. Hosts with trustworthy faces, on the other hand, more likely rent their assets with higher charges in the sharing economy domain^7^.

From a neuroscience perspective, it has been suggested that the detection of trustworthiness signals from faces may be mediated by a phylogenetically old survival circuit, which mobilizes the organism for approach and avoidance behavior in social interactions^2,8^. Critically, the amygdala, a bilateral structure from the medial lobe, seems to be involved by engaging neural systems that support attention, learning and memory processes^9–11^. Accordingly, perception of trustworthy and untrustworthy faces is associated with enhanced amygdala^3,12–14^ (for meta-analyses see^15,16^), as well as occipital and temporal cortical region activation^12^ to prioritize perceptual processing of socially salient information. This pattern of neural activity has also been found for positively and negatively valenced faces^17,18^ suggesting that the processing of cues signaling trustworthiness is rather related to general valence processing. Therefore, these findings support the emotion face overgeneralization hypothesis^2,19,20^, i.e. that responding appropriately to emotional states is such an adaptive function that prioritized processing also extends to facial cues that only resemble certain emotional expressions.

Interestingly, despite a prominent role of the amygdala in extracting subtle cues of both positive and negative faces, a negativity bias has been often reported: For instance, individuals with bilateral amygdala damage perceive untrustworthy faces as more trustworthy^21^. Consistent with this finding, greater amygdala sensitivity for untrustworthy than trustworthy faces is also reported in healthy individuals^2,12,14,22^. This suggests that processing signals of untrustworthiness may have higher adaptive value than signals of trust^2,23,24^, which is also consistent with animal and human studies showing a generally stronger defense motivation for aversive cues and contexts than for cues triggering approach motivation^2,25,26^.

Perceived untrustworthiness not only affects perception but also learning and memory processes^27,28^. When individuals learn that another individual cannot be trusted, they avoid strangers who look similar to that individual^27^. Interestingly, this avoidance pattern toward a putative untrustworthy individual is also reflected in amygdala activation. Furthermore, recent recognition memory studies using immediate testing from our own group and others suggest that the mere exposure to untrustworthy faces result in better memory for untrustworthy than for trustworthy faces^28–30^. These findings indicate that subtle cues of untrustworthiness are automatically learned and remembered. This memory advantage for untrustworthy faces may serve an adaptive function to avoid potential exploitation and harm in future social interactions.

Following up on recent recognition memory studies^28–30^, the present study addressed two open research questions: *First*, because these studies only tested recognition memory using short retention intervals, it is not known whether the memory advantage for untrustworthy faces persists after longer delays or only occurs when retrieval is tested immediately. The use of a longer retention interval would also clarify whether memory for untrustworthy information undergoes deeper consolidation or is just attributed to enhanced perceptual processing during encoding^22,31,32^ as recently suggested^30^. *Secon*d, it is widely agreed upon that recognition memory can be based on two qualitatively distinct cognitive processes: familiarity and recollection^33–35^. Familiarity is assumed to reflect a fast-acting and automatic process that is generally associated with the feeling of knowing an item without being able to recall specific details. Recollection, on the other hand, is considered a slower more elaborate memory process that includes the ability to recognize past events with additional spatial, temporal or contextual information^36^. Currently, it is not understood whether memory retrieval for untrustworthy faces is more relying on the process of familiarity or recollection. We therefore measured differences in brain potentials during viewing old and new items during a recognition memory test^36–39^. An early frontal Old/New difference (300–500 ms) is hypothesized to be sensitive to memory processes based on familiarity, while a later parietal Old/New difference (>500 msec) has been proposed as a neural correlate of recollection, as it is enhanced by depth of processing, as well as for correct source and “remember” judgments in a recognition task^39^.

In line with our behavioral study^28^ we expected to find enhanced recognition memory for untrustworthy, compared to trustworthy faces. For ERPs, two hypotheses can be assessed predicting the influence of trustworthiness on the electrophysiological indicators of familiarity and recollection: Given that prior long-term memory ERP studies reported enhanced recollection-based retrieval for motivationally relevant stimuli^37,38^, memory performance and late parietal Old/New differences may be heightened for untrustworthy, compared to trustworthy faces. Alternatively, features of trustworthiness are only subtle and help to rapidly form impressions about potential harm or joy^2^ that do not necessarily need elaborated (contextual) memory storage. Better memory for untrustworthy faces could therefore also be related to the process of familiarity, as reflected by early frontal ERP Old/New differences.

## Method

### Participants

Participants were 32 German-speaking students (6 male, 26 female; mean age: 25.1 years; 4 left-handed) from the University of Potsdam who participated for course credits or financial compensation. Based on our prior recognition memory study^28^ showing a medium-sized effect size (Cohen’s *d* = 0.57) difference in memory for untrustworthy and trustworthy faces effect size we estimated a sample size of *n* = 26 for the present study (α = 0.05; β = 0.20). Prior experience has shown a small number of drop-outs, for instance, due to bad ERP quality. We therefore increased the number of participants to the final sample size. Each individual provided written informed consent for a protocol approved by the Review Board of the German Psychological Society and in accordance with the Declaration of Helsinki. All participants had normal or corrected-to-normal vision.

### Stimuli

The stimulus material consisted of 120 neutral faces with direct gaze, which were previously evaluated as high (30 female, 30 male faces) and low (30 female, 30 male faces) in trustworthiness by an independent sample (see^31^ for details on set construction). Following an established procedure^40^, the faces were converted into grayscales, equalized in size, position and luminance and surrounded by an elliptic mask using Adobe Photoshop CS4 (Adobe Systems Inc., San Jose, CA) and Matlab 7.7 (MathWorks Inc., Natick, MA) to minimize the influence of expression-irrelevant features on face processing during the present task.

### Procedure

The study consisted of two experimental sessions: an encoding session and a recognition memory session one week later. The sessions took place in a sound-attenuated dimly lit room. Procedure and stimuli were the same as in our prior studies^28,31^. Participants viewed 60 faces (30 trustworthy and 30 untrustworthy faces) in pseudorandom order. Participants were instructed to pay attention to the faces but were not informed that the faces differed in trustworthiness and that a recognition test would follow (incidental encoding). Each face was presented once for 3,000 ms, followed by an intertrial interval (ITI) of 5,500, 6,000, or 6,500 ms. A fixation cross was present in all faces trials and ITIs to ensure that participants fixated the center of the screen.

One week later, participants returned to the lab for the recognition memory session. After the electrode net was placed on the head, participants viewed the 60 old faces together with 60 (30 trustworthy and 30 untrustworthy faces) new faces in pseudorandom order. Each trial started with the presentation of a face for 3,000 ms. After picture offset, the question “Old/New?” was presented and participants had to decide whether the face had been viewed before during encoding (i.e., by pressing “Old”) or not (i.e., by pressing “New”). Responses were made on two different buttons on a keyboard and response buttons were counterbalanced across participants. The assignment of the faces to the old and new face set was counterbalanced across participants. After the recognition memory task, the participants rated one of the two sets (i.e., their encoding set) for subjective trustworthiness using a similar rating task as in our previous studies^28,31^. In this task, participants were encouraged to rely on their feelings and to rate the trustworthiness of the faces as fast as possible on a rating scale, ranging from 1 (*untrustworthy*) to 9 (*trustworthy*). In line with our prior work^28,31^, analyses of these ratings revealed that the preselected untrustworthy faces were rated as less trustworthy (M = 4.36; SD = 1.21) than the preselected trustworthy faces (M = 5.89; SD = 0.96), *t*(31) = 12.64, *p* < 0.001, confirming the validity of the face set (see also supplementary information for further analysis showing significant differences in trustworthiness when controlled for face typicality and distinctiveness).

### Electrophysiological recording

Electrophysiological signals were continuously recorded from 129-sensor nets using an Electrical Geodesic system (EGI, Eugene, OR) and digitized at a rate of 250 Hz, using the vertex sensor (Cz) as recording reference. Scalp impedance of all channels was kept below 50 kΩ, as recommended by the manufacturer’s guidelines. Online, all channels were bandpass filtered (0.1–100 Hz). Offline, ElectroMagnetic EncaphaloGraphy software (EMEGS^41^) was used to preprocess the data for subsequent analyses, including low-pass filtering (40 Hz), artifact detection, sensor interpolation, baseline correction, and conversion to an average reference^42^. The MATLAB-based toolbox BioSig^43^ was used for eye movement and blink artifacts corrections of the extracted epochs. This method is based on linear regression to reliably remove electrooculogram activity from the EEG^44^. Stimulus-synchronized epochs were extracted from 100 ms before to 1,200 ms after face onset and baseline corrected (100 ms prior to stimulus onset).

### Data analysis

ERPs were computed for each sensor and participant. Only trials with correct responses were included in ERP averages. In consideration of previous research showing distinct ERP old/new effects^37,39,45,46^ and based on visual inspection of the waveforms, mean ERP amplitudes were analyzed in an early window from 350 to 550 ms over frontal regions (EGI sensors: 5, 9, 10, 11, 12, 15, 16, 18, and 22), and in a late window from 550 to 750 ms over a centro-parietal cluster (EGI sensors: 7, 31, 55, 80, 106, and 129), where the difference between old and new conditions was maximal. To examine the effects of trustworthiness on recognition-related ERPs, data were analyzed in a two-way ANOVA using the factors *Memory* (old, new) and *Trustworthiness* (untrustworthy, trustworthy) as repeated measures for the early and late time window, separately. To note, we particularly focused on ERPs related to explicit recognition memory. ERP analyses related to face processing and repetition^47^ did not reveal any trustworthiness x memory interactions and were provided as supplementary information. Furthermore, ERP analyses related to face processing during encoding showing that the main findings of this study are not driven by differences in distinctiveness between trustworthy and untrustworthy faces (e.g., P200)^47^ were also included as supplementary information.

For behavioral performance, hit rate (H), false alarm rate (FA), recognition accuracy (Pr = H−FA), and response bias [Br = *p*(FA)/*p*(1 − Pr)] as recommended by Snodgrass and Corwin (two-high-threshold model)^48^ were analyzed for trustworthy and untrustworthy faces. These indices were complemented by analyses of detection and bias parameters derived from signal detection theory (d′ = *z*(H) − *z*(FA) and C = −0.5[(*z*(H) + *z*(FA)]). T-tests for dependent means were computed for each of the behavioral indices to test for significant differences. All statistical analyses were conducted using IBM SPSS Statistics 24 and JMP 5.0.

## Results

### Behavioral performance

Table 1 lists participants’ memory performance for trustworthy and untrustworthy faces. Hit rates did not differ between untrustworthy und trustworthy faces (*t*(31) < 1). However, new untrustworthy faces were more easily recognized than new trustworthy faces, as indicated by significantly lower false alarm rates, *t*(31) = 3.56, *p* = 0.001. As expected, recognition accuracy was higher for untrustworthy than for trustworthy faces as shown by larger *Pr*, *t*(31) = 3.41, *p* = 0.002, and d′ values, *t*(31) = 2.75, *p* = 0.01. Response bias for faces did not differ as a function of trustworthiness as indicated by similar *Br*, *t*(31) = 1.24, *p* = 0.23, and C values, *t*(31) < 1. Taken together, memory performance was better for untrustworthy than trustworthy faces when tested after a one week retention interval.

**Table 1:** Recognition memory performance.

	Trustworthy faces	Untrustworthy faces
Hits (H)	0.59 (0.15)	0.61 (0.14)
False Alarms (FAs)	0.38 (0.17)	0.32 (0.16)*
**Discrimination indices**		
Pr	0.21 (0.15)	0.29 (0.18)*
d′	0.61 (0.50)	0.82 (0.56)
**Response bias indices**		
Br	0.47 (0.20)	0.44 (0.17)
C	0.07 (0.44)	0.11 (0.35)

Numbers in parentheses indicate SD. Higher *Pr* and *d*′ values indicate better discriminability between old and new faces. *Br* values higher than 0.5 indicate liberal response criteria; lower *Br* values suggest conservative response bias. Negative *C* values correspond to a liberal response bias; positive *C* values correspond to a conservative response bias. *p < 0.01.

### ERPs

#### Frontal Old/New effect reflecting familiarity

**Figure 1:**
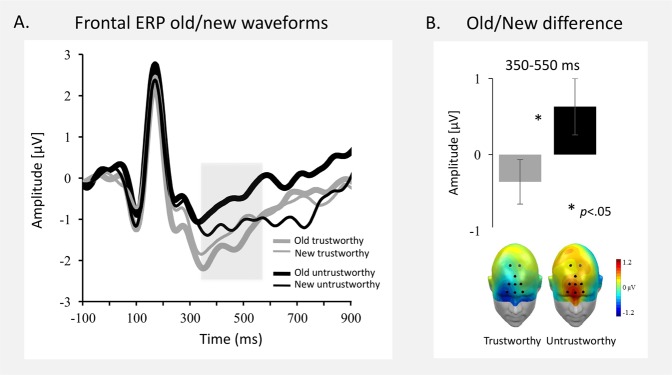
Frontal ERP Old/New effect reflecting familiarity. (**A**) Grand-averaged waveforms at representative frontal cluster for correctly recognized old and new trustworthy and untrustworthy faces. The shaded area represents the early (350–550 ms) time window used for the analyses. (**B**) ERP Old/New difference of the mean amplitudes and scalp topographies of the ERP difference (350–550 ms) for trustworthy and untrustworthy faces.

Figure 1A illustrates the grand average ERPs for correctly recognized old and new faces that differed in trustworthiness collapsed across a representative frontal sensor cluster.

At frontal regions, in the early time window (350–550 ms), a main effect of the factor *Trustworthiness* was found, *F* (1,31) = 6.90, *p* = 0.013, *η*_p_^2^ = 0.18, which interacted with the factor *Memory*, *F* (1,31) = 4.47, *p* = 0.043, *η*_p_^2^ = 0.13. Follow-up testing revealed that the Old/New difference was larger for untrustworthy than for trustworthy faces, *t*(31) = 2.11, *p* = 0.043 (see Fig. 1B). In the later time window (550–750 ms), the ERP old/new difference continued, *Memory*: *F* (1,31) = 5.71, *p* = 0.023, *η*_p_^2^ = 0.16, but the interaction with *Trustworthiness* only reached trend level, *F* (1,31) = 3.75, *p* = 0.062, *η*_p_^2^ = 0.11. *Trustworthiness* did not reach significance (*F* < 1).

#### Parietal Old/New effect reflecting recollection

**Figure 2:**
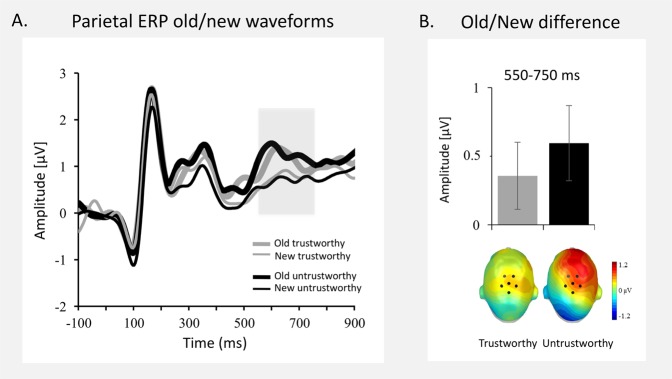
Centro-parietal ERP Old/New effect reflecting recollection. (**A**) Grand-averaged waveforms at representative centro-parietal cluster for correctly recognized old and new trustworthy and untrustworthy faces. The shaded area represents the late (550–750 ms) time window used for the analyses. (**B**) ERP Old/New difference of the mean amplitudes and scalp topographies of the ERP difference (550–750 ms) for trustworthy and untrustworthy faces.

Figure 2A illustrates the grand average ERPs for correctly recognized old and new faces that differ in trustworthiness collapsed across a representative centro-parietal sensor cluster.

No significant main effects or interactions (all *F*s < 3.19; *p* > 0.084) were observed at centro-parietal regions in the early time window (350–550 ms). However, in the time window between 550 and 750 ms, old faces prompted larger ERP positivity than new faces, as reflected by a main effect of *Memory*, *F* (1,31) = 6.49, *p* = 0.016, *η*_p_^2^ = 0.17 (see Fig. 2B). No other main effects or interactions reached significance in this later time window (all *F*s < 1).

Taken together, untrustworthy faces generated larger early ERP old/new effects over frontal electrodes than trustworthy faces, indicating facilitated familiarity-based recognition. No such differences were observed for the recollection-sensitive late centro-parietal ERP Old/New effect.

## Discussion

In the present study, ERPs were measured to assess the effects of facial trustworthiness on long-term memory. Consistent with prior behavioral studies using immediate recognition tests^28–30^, better memory discrimination was found for untrustworthy than for trustworthy faces after a one week retention interval. During memory retrieval, correctly recognized old faces evoked enhanced ERP positivity, relative to unseen novel faces. This ERP Old/New difference was most pronounced for untrustworthy faces over frontal electrode sites in an early 350–550 ms window. The results provide behavioral and neural evidence for a long-lasting memory advantage for untrustworthy faces.

Using ERPs, we were able to assess how memory for trustworthy and untrustworthy faces is formed and retrieved. In line with previous studies^36,37,39^, we found enhanced ERP positivity for old, relative to new items with specific spatio-temporal characteristics. While the late ERP Old/New effect reflecting the memory process of successful recollection did not vary as a function of trustworthiness, we found that particularly untrustworthy faces modulated the early Old/New effect, a putative correlate of familiarity-based remembering^49^ (which may also involve implicit memory processes^50^). According to dual-process theory familiarity has been considered as a fast-acting, less effortful and relatively automatic memory process that works with greater efficiency than recollection^35,51^. While familiarity supports simply knowing that an event was encountered before, recollection is understood as retrieving additional (contextual) details (e.g., temporal, spatial, situational) that are associated with an event. For instance, prior ERP studies (for review see^36^) found that the early frontal old/new effect, compared to the late parietal old/new effect, is insensitive by depth of processing^52^ and divided attention^53^ during encoding. Furthermore, the early frontal old/new effect varies with familiarity strength^54^ and memory confidence^37^, the parietal old/new effect, on the other hand is specific for recollection (e.g., sensitive to the amount of information recollected^55^, correct source judgments^56^ and recollection based Remember judgments^57^ and high confidence memory judgments^37^). The ERP finding that recognition of untrustworthy individuals is based on familiarity (reflected by early frontal old/new differences) fits with the emotion overgeneralization hypothesis^2,20^, which posits that face-based valence judgments are an adaptive mechanism, which helps in a rapid and efficient way to avoid upcoming harm. From this view, the potential cost of responding with approach behavior to an untrustworthy individual is higher than the potential cost of avoiding a trustworthy individual. This also implies that untrustworthy faces do not necessarily need to be stored in a more complex contextual fashion (e.g., recollection) when the evolutionary purpose is to quickly recognize socially relevant information. Interestingly, contrary to prior ERP studies using emotional scenes^38,58^ or faces^45^, we did not find a modulation in the recollection-sensitive parietal old/new effect. However, emotional scenes and faces contain more contextual feature information and draw more attention^59,60^ than neutral faces, which may have resulted in enhanced recollection-specific contextual binding^61^ and enhanced parietal ERP old/new differences in these studies.

Enhanced acontextual familiarity-based memory for untrustworthy faces may also have serious consequences on social decision making. For instance, previous lab and more real life studies found that individuals whose faces are perceived as untrustworthy are less trusted^62,63^, are less likely to have their loans funded^5^ and are more likely to be sentenced for crimes^6^. Given that face-based social inferences about a person’s trustworthiness are not necessarily accurate and reliable in general^19^, familiarity-based remembering may come at costs. On the one hand, with limited additional (reputational) information about a person, individuals can instantaneously form first impressions that are stored in a less contextual manner (which likely engage less processing resources) that may help for quick decision making whether a person can be trusted or not (irrespective of its validity). Memory for untrustworthy faces may in turn also affect other social preferences for fairness and cooperation and contribute to less prosocial behavior and emotions, including altruism^64^ and empathy^65^. On the other hand, familiarity-based memory for untrustworthy faces may even more strongly hinder to derive valid appearance-based impressions about other persons. Social decision making strongly relies on available contextual information (e.g., reputational information, social context etc., see^66^). Recognizing an untrustworthy face including remembrance of such contextual information may therefore also be adaptive (in the long term) in a sense that it helps to correct invalid appearance-based judgments. It must be noted, however, that our study was intended to investigate long-term recognition memory for trustworthy and untrustworthy looking faces that were incidentally encoded once, mimicking a brief social encounter in real life^29^. Future research need to explore whether additional reputational information and increased experience (e.g. through repetition of trials) may also enhance recollection memory.

Finally, we can only speculate about the underlying neural mechanism leading to enhanced familiarity-based remembering for untrustworthy faces in the present study. As suggested by Tsukiura^67^, valence-based memory for faces is mediated by the amygdala, which detects emotional properties of the faces (such as conveyed by subtle cues^2^) and interacts with the medial orbitofrontal cortex (OFC) and insular cortex (IC) to process positive and negative signals, respectively. These regions in turn may modulate a network consisting of hippocampus (HC) and fusiform faces area to enhance memory for these faces depicting affective signals^67,68^. Given that memory for untrustworthy faces was mediated by familiarity, it is likely, however, that the perirhinal cortex (PrC) rather than the HC, contribute to the memory-enhancing effect since the PrC has been specifically related representing item familiarity^69–71^.

Taken together, we found that the memory advantage for faces signaling untrustworthiness is remarkably stable over time. In support of the overgeneralization hypothesis, we found electrophysiological evidence that memory for untrustworthy faces is likely linked to familiarity-based recognition, a fast, less specific and automatic retrieval process, which helps to quickly recognize those who may cause potential harm in social interactions.

## Supplementary information

Behavioral and neural evidence of enhanced long-term memory for untrustworthy faces
