# Supplementary material for: Behavioral and neural evidence of enhanced long-term memory for untrustworthy faces
Source: Sci Rep. 2019 Dec 16;9:19217. doi: 10.1038/s41598-019-55705-7 (PMC6915708; doi:10.1038/s41598-019-55705-7)
Supplement: Supplementary file 1 — Behavioral and neural evidence of enhanced long-term memory for untrustworthy faces [file 41598_2019_55705_MOESM1_ESM.pdf]

Behavioral and neural evidence of enhanced long-term memory for untrustworthy faces

Mathias Weymar[1], Carlos Ventura-Bort[1], Julia Wendt[1,2], & Alexander Lischke[2]

[1]Department of Psychology, University of Potsdam, Germany

[2]Department of Biological and Clinical Psychology, University of Greifswald, Germany

Supplementary Information

Method

Rating Task.

During the reviewing process of this paper, we recruited 10 participants (4 women) to evaluate whether face typicality and distinctiveness is related to perceived trustworthiness. We therefore asked the participants to rate both face sets for subjective trustworthiness (see for procedure[1]), distinctiveness and typicality. Distinctiveness was obtained by the face-in-the-crowd (FITC) measure by Valentine and Bruce[2] and typicality was obtained by an adapted version of the deviation ratings used by Wickham and Morris[3], see also Wiese and colleagues[4]. For the FITC, participants were instructed to rate distinctiveness on a scale between 1 (lowly distinct) and 9 (highly distinct) by asking themselves how easily they would spot the face in a group of people (e.g. a crowded platform). For typicality, participants were asked to rate the extent to which the presented faces deviated from other faces that they know on a scale between 1 (very typical) and 9 (very atypical).

Rating results.

We found that the preselected untrustworthy faces (M= 5.5; SD = 0.47) were rated as less trustworthy than the preselected trustworthy faces (M= 7.01; SD = 0.34), $t(9) = 13.91$, $p <$ .001. Furthermore, untrustworthy faces were also perceived as less typical (M= 6.65; SD =

0.67), in line with Todorov and colleagues[4], and more distinct (M= 6.37; SD = 0.59) than trustworthy faces (typicality: M= 5.59; SD = 0.57, $t(9) = 6.64$, $p< .001$; distinctiveness: M= 5.19; SD = 0.45, $t(9) = 7.01$, $p< .001$). However, when controlling for typicality and distinctiveness (included as Covariates), there was still a difference in trustworthiness of the faces ($F_{1,7}= 18.88$, p= .003) indicating that perceived trustworthiness of the faces cannot be fully explained by face typicality and distinctiveness.

ERP data analysis.

To examine the effects of face processing and typicality/distinctiveness[5,6] during encoding, as well as face processing and repetition during recognition[6,7] we analyzed the N170, P200, and the N250. In consideration of previous research and based on visual inspection of the waveforms, the N170 was analyzed in the time window from 145 to 185 ms, the P200 was analyzed in the time window between 220 to 270 ms, and the N250 was analyzed in the time window from 270 to 400 ms, using the following parietal EGI sensor clusters (N170 and N250: 58, 63, 64, 65, 68, 69, 70, 83, 89, 90, 94, 95, 96, and 99; P200: 62, 67, 71, 72, 76 and 77). ERP data were analyzed using a dependent t-test comparing processing of trustworthy and untrustworthy faces during encoding, and in a two-way ANOVA using the factors *Memory* (old, new) and *Trustworthiness* (untrustworthy, trustworthy) as repeated measures during recognition, for the N170, P200 and N250, separately.

ERP results

Encoding: N170, P200 and N250

For this encoding analysis two participants were not included due to poor EEG quality and technical problems during recording. Analysis of the N170 (145 – 185 ms), P200 (220 – 270 ms) and N250 (270 – 400 ms) revealed no differences between trustworthy and untrustworthy faces (all t (29) < 1.) indicating that face processing was not influenced by

trustworthiness. Critically, typicality has been strongly related to variations in the P200 (see also for N250[5]) with usually smaller P200 amplitudes for distinctive relative to more typical faces. Both components, however, showed no differences between trustworthy and untrustworthy faces (for P200 see Supplementary Figure below) suggesting that the main findings were not confounded by specific face characteristics, such as typicality and distinctiveness.

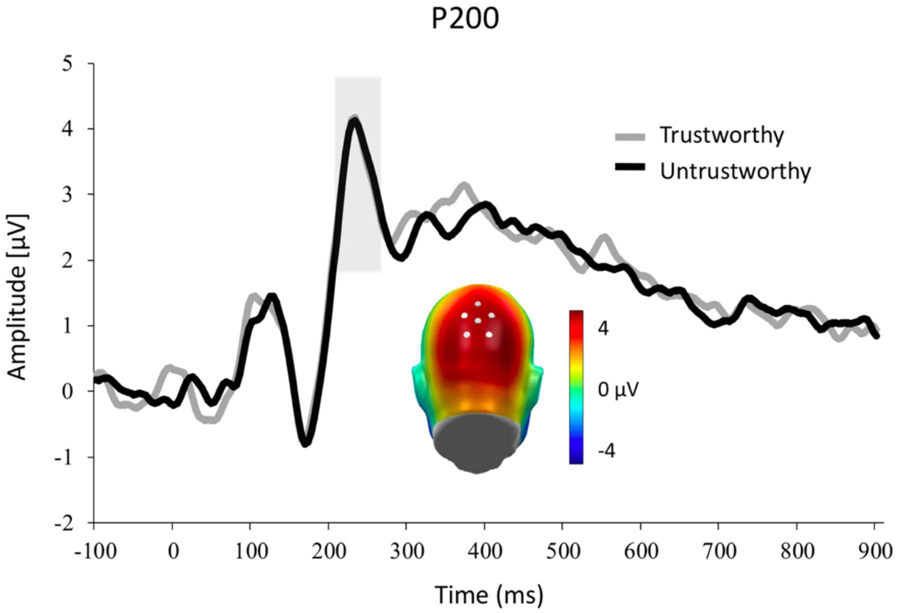

Supplementary Figure. Typicality sensitive P200 as a function of trustworthiness. Grand-averaged waveforms at representative parietal cluster for trustworthy (gray line) and untrustworthy (black line) faces. The shaded area represents the (220-270 ms) time window used for the P200 analyses. The inset shows the scalp topography of the P200 maximum irrespective of trustworthiness for the selected time window and sensor cluster.

Recognition: N170, P200 and N250

During recognition, analysis of the N170 (145 – 185 ms) revealed no significant main effects of *Trustworthiness:* (F (1,31) < 1), *Memory* (F (1,31) = 2.01, p = .17) and interactions (*Trustworthiness* x *Memory*: F (1,31) < 1).

As for the N170, no main effects and interactions (*Trustworthiness:* (*F* (1,31) = 2.15, *p* = .15); *Memory* (*F* (1,31) < 1); *Trustworthiness* x *Memory*: *F* (1,31) = 2.84, *p* = .10) were observed in the later time window (220 - 270 ms) for the P200.

For the N250, in the time window between 270 to 400 ms, a main effect of the factor *Trustworthiness* was found, *F* (1,31) = 6.80, *p* = .014. No effects of Memory and interactions with *Trustworthiness*, however, were observed for this component (all *F* < 1).

Taken together, different ERP components related to face processing during recognition did not reveal any memory related effects of *Trustworthiness* during recognition. It must be noted, however, that the N250 findings are in contrast to prior research[6,8] linking the N250 to face familiarity (e.g., own faces vs. unfamiliar face) and repetition (repeated vs. unfamiliar faces). Particularly, the lack of a repetition effect in our study may be explained by the smaller amount of repetitions in our study (we only presented a face *once*). Prior research found that the N250 needs higher number of learning trials to differentiate between repeated and familiar faces[8].

References

1. Wendt, J., Weymar, M., Junge, M., Hamm, A. O. & Lischke, A. Heartfelt memories: Cardiac vagal tone correlates with increased memory for untrustworthy faces. *Emotion* **19**, 178-182 (2019).
2. Valentine, T. & Bruce, V. The effects of distinctiveness in recognizing and classifying faces. *Perception* **15**, 525–535 (1986).
3. Wickham, L. H. V. & Morris, P. E. Attractiveness, distinctiveness, and recognition of faces: Attractive faces can be typical or distinctive but are not better recognized. *Am. J. Psychol.* **116**, 455–468 (2003).

4. Todorov, A., Olivola, C. Y., Dotsch, R. & Mende-Siedlecki, P. Social attributions from faces: Determinants, consequences, accuracy, and functional significance. *Annu. Rev. Psychol.* **66**, 519-545 (2015).
5. Schulz, C. Kaufmann, J. M., Kurt, A. & Schweinberger, S. R. Faces forming traces: Neurophysiological correlates of learning naturally distinctive and caricatured faces. *Neuroimage* **63**, 491-500 (2012).
6. Wiese, H., Altmann, C. S. & Schweinberger, S. R. Effects of attractiveness on face memory separated from distinctiveness: Evidence from event-related brain potentials. *Neuropsychologia* **56**, 26-36 (2014).
7. Wiese, H., Schweinberger, S. R. & Hansen, K. The age of the beholder: ERP evidence of an own-age bias in face memory. *Neuropsychologia* **46**, 2973-2985 (2008).
8. Tanaka, J.W., Curran, T., Porterfield, A.L. & Curran, T. Acquisition of pre-existing and acquired face representations: the N250 ERP as an index of face familiarity. *J. Cogn. Neurosci.* **18**, 1488–1497 (2006).
